# Evaluation of a Methylated Circulating Tumour DNA Panel for Detection and Disease Stratification in Prostate Cancer

**DOI:** 10.3390/ijms27136081

**Published:** 2026-07-07

**Authors:** Stine V. Eriksen, Ahmed H. Zedan, Søren Kahns, Palle J. S. Osther, Signe Timm, Torben F. Hansen

**Affiliations:** 1 Department of Oncology, Vejle Hospital, University Hospital of Southern Denmark, 7100 Vejle, Denmark; ahmed.zedan@rsyd.dk (A.H.Z.); signe.timm@rsyd.dk (S.T.); torben.hansen@rsyd.dk (T.F.H.); 2 Department of Regional Health Research, Faculty of Health Sciences, University of Southern Denmark, 5230 Odense, Denmark; 3 Department of Biochemistry and Immunology, Vejle Hospital, University Hospital of Southern Denmark, 7100 Vejle, Denmark; soren.kahns@rsyd.dk; 4 Department of Urology, Vejle Hospital, University Hospital of Southern Denmark, 7100 Vejle, Denmark; palle.joern.osther@rsyd.dk

**Keywords:** prostate cancer, circulating tumour DNA, ctDNA methylation, liquid biopsy, droplet digital PCR

## Abstract

Prostate cancer (PCa) exhibits marked biological heterogeneity, complicating accurate identification of aggressive disease at initial diagnosis. This study investigated whether circulating tumour DNA (ctDNA) methylation markers enhance detection and risk stratification across the PCa disease continuum. Plasma samples from the initial hospital visit were obtained from 280 participants in the PerPros prostate biobank (Vejle, Denmark), all referred with suspected PCa. Following diagnostic work-up, participants were classified across the PCa disease spectrum or as biopsy-confirmed PCa-free controls. Five methylated CpG regions (*DOCK2*, *HAPLN3*, *ACTRT2*, *EVX1*, *HOXD13*) were analysed using multiplex ddPCR. Performance was assessed using ROC analysis and logistic regression and compared with prostate-specific antigen (PSA). Methylation markers were detectable across the PCa disease spectrum, most frequently in metastatic castration-sensitive PCa (mCSPC) (85/97; 88%). The ctDNA methylation panel discriminated mCSPC from controls with an AUC (area under the curve) of 0.88 (95% CI: 0.83–0.93). In this cohort, combining PSA (cut-off 20 µg/L) with the ctDNA methylation panel significantly improved discrimination between patients with mCSPC and locally advanced PCa compared with PSA alone (AUC 0.88; 95% CI 0.83–0.94; *p* < 0.001) vs. 0.78 (95% CI 0.71–0.84). In contrast, discrimination between localised/locally advanced PCa and patients with biopsy-confirmed controls was limited (AUC 0.56; 95% CI: 0.51–0.61). The ctDNA methylation panel demonstrates robust identification of metastatic PCa and can effectively differentiate mCSPC from locally advanced PCa. However, its diagnostic abilities in localised disease appear limited.

## 1. Introduction

Prostate cancer (PCa) is the second most diagnosed malignancy among men worldwide, with over 1.4 million new cases each year [1]. Due to the wide variability in disease presentation, ranging from indolent to highly aggressive PCa, outcomes vary markedly across the disease spectrum. Patients diagnosed with localised PCa generally have long-term survival, whereas those presenting with de novo metastatic disease have a substantially poorer prognosis [2,3]. These differences are driven mainly by the extent of disease at diagnosis. However, considerable heterogeneity in clinical outcomes has also been observed among patients with the same disease stages, reflecting underlying biological and molecular diversity [4]. Consequently, accurate diagnostic and risk stratification tools are essential for more personalised management, including decisions about escalation and de-escalation of therapy.

Diagnostic assessment of PCa follows a stepwise approach, typically initiated by an elevated prostate-specific antigen (PSA) level. Owing to its limited specificity, PSA is insufficient to warrant prostate biopsy and requires interpretation alongside clinical findings and additional diagnostic modalities [5]. Moreover, aggressive disease may occur despite normal PSA levels [6].

Imaging-based modalities, including magnetic resonance imaging (MRI), have improved the triage of men with suspected clinically significant PCa (csPCa) and patient selection for biopsy [5]. Although pre-biopsy MRI reduces the detection of clinically insignificant PCa, overdiagnosis and overtreatment remain clinical challenges [7,8,9,10].

Risk stratification nomograms have been developed over the past three decades [11,12]; however, their widespread adoption remains limited by insufficient external validation, the inherent heterogeneity of PCa, and the pitfalls of traditional diagnostic tools [13,14]. Therefore, there has been sustained interest in incorporating more specific biomarkers to complement established clinical and imaging strategies [5,15,16].

Circulating tumour DNA (ctDNA) offers a minimally invasive assessment of tumour-derived genetic and epigenetic alterations across the overall tumour burden [17]. Aberrant CpG-island methylation is an early and frequent feature of prostate carcinogenesis, making it an appealing target for liquid biopsy-based assays [18,19]. Methylation-based ctDNA assays use a tumour-agnostic approach, relying on recurrent PCa-associated methylation patterns, avoiding the need for patient-specific mutations required in tumour-informed assays. Previous studies have demonstrated that PCa-associated methylation changes are detectable in the circulation, supporting the feasibility of ctDNA methylation analysis as a biomarker strategy [19,20,21]. Nevertheless, ctDNA detection in localised PCa remains challenging due to low ctDNA fractions in early-stage disease. In particular, studies by Bjerre et al. [20] and Dillinger et al. [21] have shown that tumour-associated methylations can be reliably measured in plasma and correlate with tumour burden. The role of methylated ctDNA in improving csPCa identification remains underexplored, particularly in the early stages of PCa, where mutation-based assays have limited sensitivity.

This study aimed to evaluate the performance of a ctDNA methylation panel in both diagnostic settings and in risk stratification for patients diagnosed with PCa.

## 2. Results

### 2.1. Participant Characteristics

A total of 280 participants from the PerPros Biobank at Vejle Hospital were included: controls (*n* = 65), patients diagnosed with low-risk PCa managed by active surveillance (AS) (*n* = 55), patients diagnosed with locally advanced PCa treated with radical prostatectomy (RP) (*n* = 63), and patients presenting with de novo metastatic castration-sensitive PCa (mCSPC) (*n* = 97). Patients with mCSPC were older (median age 72 years) and had higher baseline PSA levels (median PSA 84 µg/L) than patients in the other three groups. In the control group, 29% of men had PSA > 10 µg/L. In the mCSPC group, 86.7% of patients and 47.6% in the RP group had csPCa (defined as grade group ≥ 3, according to International Society of Urological Pathology (ISUP)) [22]. Among patients with mCSPC, 22% had visceral organ metastases at baseline, and 72% had high tumour volume as defined by the CHAARTED study [23]. In the RP cohort, 37% had pathologically confirmed N1 disease. Baseline characteristics are presented in Table 1.

### 2.2. Assay Detection and Limit of Blank

**Figure 1:**
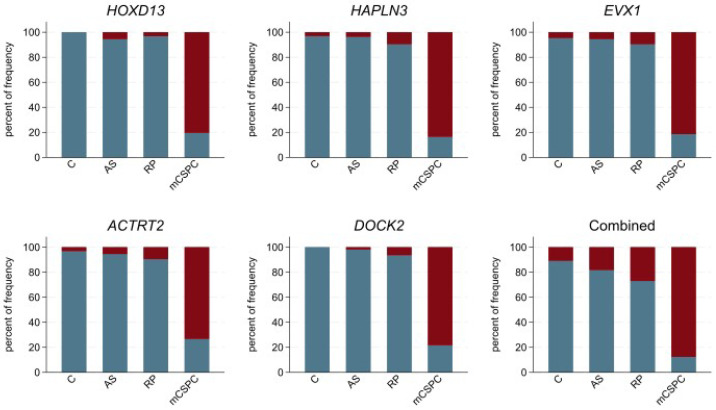
Detection of individual methylation markers and the combined ctDNA methylation panel across clinical groups. Distribution of circulating tumour DNA (ctDNA) positivity across controls, active surveillance, radical prostatectomy, and metastatic castration-sensitive prostate cancer. Red indicates ctDNA-positive samples and blue indicates ctDNA-negative samples. *n* = 65 (C), 55 (AS), 63 (RP), 97 (mCSPC). C: controls; AS: active surveillance; RP: radical prostatectomy; mCSPC: metastatic castration-sensitive prostate cancer.

Assay-specific limits of blank (LoB) with 95% CI were confirmed in plasma from controls (*n* = 65) [24]. The LoB was zero droplets for *DOCK2*, one droplet for *HAPLN3*, two droplets for *HOXD13* and *EVX1*, and five droplets for *ACTRT2*. These values were consistent with previous reports, except for *EVX1*, which is one droplet higher (i.e., two droplets). Samples were classified as ctDNA-positive when droplet counts exceeded marker-specific LoBs. Panel performance was profiled across thresholds from ≥1 to 5 positive markers (Figure S1; Table S1). For subsequent analyses, the panel was defined as positive if ≥1 marker was detected (data-derived in this cohort), as this threshold yielded the highest combined sensitivity (52.1%) and specificity (89.2%). The discriminative ability of the marker count corresponded to an AUC of 0.73 (95% CI: 0.68–0.77). Positivity rates for the individual methylation markers across the four groups ranged from 0 to 4.6% in controls, 1.8 to 5.5% in the AS group, 3.2 to 9.5% in the RP group, and 73.2 to 83.5% in the mCSPC group. Positivity for the combined panel (≥1 positive marker) was 10.8% in controls, 18.2% in the AS group, 27.0% in the RP group, and 87.6% in the mCSPC group (Figure 1).

### 2.3. Diagnostic Performance of Methylation Markers

**Figure 2:**
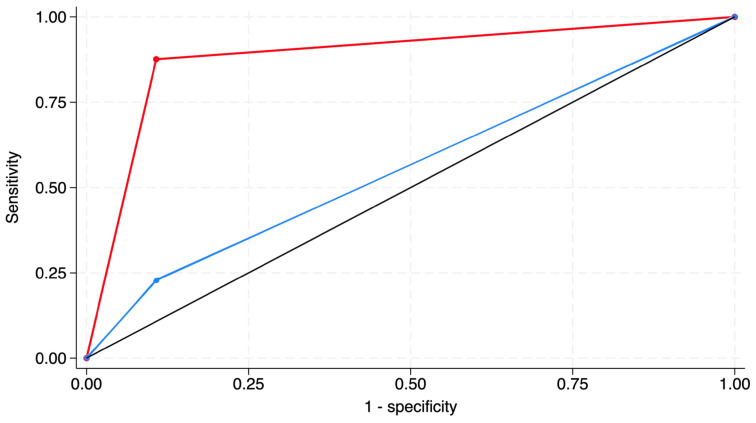
Diagnostic performance of the ctDNA methylation panel for discrimination of metastatic and localised prostate cancer from controls. Receiver operating characteristic (ROC) curves for discrimination of metastatic castration-sensitive prostate cancer from controls, AUC: 0.88 (95% CI: 0.83–0.93) (red), and localised/locally advanced prostate cancer (active surveillance and radical prostatectomy) from controls, AUC: 0.56 (95% CI: 0.51–0.61) (blue). ROC analyses were based on the combined panel positivity threshold of ≥1 positive marker. *n* = 97 vs. 65 (mCSPC vs. controls); 118 vs. 65 (localised disease vs. controls). AUC: area under the curve; ctDNA: circulating tumour DNA; C: controls; AS: active surveillance; RP: radical prostatectomy; mCSPC: metastatic castration-sensitive prostate cancer.

ROC analyses were conducted to assess the discriminative ability of both the individual methylation markers and the ctDNA methylation panel between controls and patients diagnosed with PCa across the three groups, namely, AS, RP, and mCSPC (Table 2, Figure S2). The ctDNA methylation panel discriminated patients with PCa from controls with an AUC of 0.71 (95% CI: 0.66–0.76) (Figure S2). Diagnostic performance using baseline PSA levels of 5, 10, and 20 µg/L showed AUC values of 0.53, 0.65, and 0.68, respectively (Figure S3, Table S2). In subgroup analyses, the AUC for the ctDNA methylation panel in the mCSPC group versus controls was 0.88 (95% CI: 0.83–0.93), and in localised/locally advanced PCa (both AS and RP) versus controls, it was 0.56 (95% CI: 0.51–0.61) (Figure 2; Table S3). Sensitivity, specificity, PPV, and NPV are reported in Table S3.

### 2.4. Clinical Performance of the ctDNA Methylation Panel for Risk Stratification of Patients with PCa: Active Surveillance Versus Active Treatment Groups

In patients diagnosed with PCa across the three groups (AS, RP, and mCSPC), the ability of the ctDNA methylation panel to discriminate between clinically distinct risk groups was investigated. Using the ctDNA methylation panel, discrimination between patients in the AS group and patients in both the RP and mCSPC groups yielded an AUC of 0.73 (95% CI: 0.66–0.79) (Figure S4A). Among patients diagnosed with local/locally advanced PCa, discrimination between those managed with AS and those managed with RP using the ctDNA methylation panel yielded an AUC of 0.54 (95% CI: 0.47–0.62) (Figure S4B). Sensitivity, specificity, PPV, and NPV are reported in Table S3.

### 2.5. Discrimination Across Patients Managed by Active Management: RP Versus mCSPC

**Figure 3:**
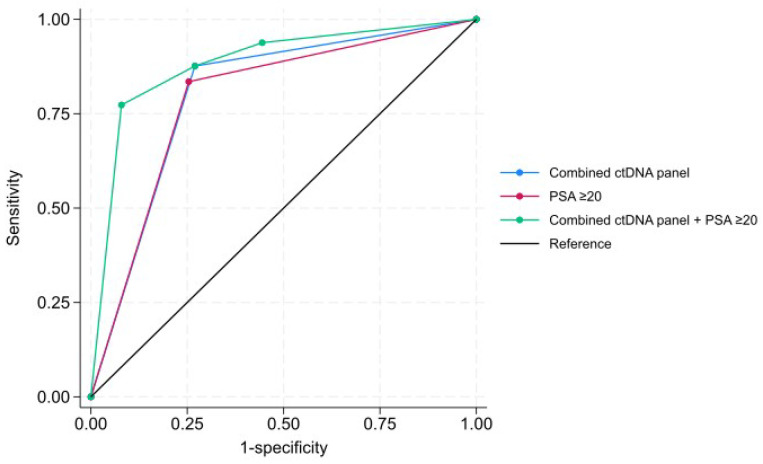
ROC curves for ctDNA and PSA in discriminating metastatic castration-sensitive prostate cancer from locally advanced prostate cancer treated with radical prostatectomy. Receiver operating characteristic (ROC) curves comparing a ctDNA methylation panel, PSA ≥ 20 µg/L, and their combination in patients with mCSPC (*n* = 97) and locally advanced disease (*n* = 63). ctDNA: circulating tumour DNA; PSA: prostate-specific antigen; RP: radical prostatectomy; mCSPC: metastatic castration-sensitive prostate cancer.

ROC analyses were performed to assess discrimination between patients managed by RP and those diagnosed with mCSPC using the ctDNA methylation panel. The ctDNA methylation panel had an AUC of 0.80 (95% CI: 0.74–0.87) (Figure 3). An AUC of 0.88 (95% CI: 0.83–0.94) was observed for the ctDNA methylation panel and PSA (20 µg/L), compared with PSA alone, with an AUC of 0.78 (95% CI: 0.71–0.84) (*p* = 0.0003), as shown in Figure 3. Sensitivity, specificity, PPV, and NPV are reported in Table S3.

### 2.6. Multivariable Analysis of ctDNA and Clinicopathological Variables

To explore whether the ctDNA methylation panel provided incremental information beyond established clinicopathological variables, exploratory multivariable logistic regression analyses were performed comparing patients with mCSPC (*n* = 97) and patients with localised and locally advanced PCa (AS and RP combined; *n* = 118). Addition of the ctDNA methylation panel significantly improved model fit compared with models including PSA alone (LR χ^2^ = 21.7, *p* < 0.001), PSA and ISUP grade (LR χ^2^ = 13.2, *p* = 0.0003), and PSA, ISUP grade, and T-stage (LR χ^2^ = 11.5, *p* = 0.0007). In the fully adjusted model, ctDNA methylation panel positivity remained associated with metastatic disease (OR 8.5, 95% CI 2.3–31.0, *p* = 0.001).

## 3. Discussion

The ctDNA methylation panel was evaluated for diagnostic and risk stratification performance across the PCa spectrum. Four principal findings were observed. First, methylation markers were detectable across disease stages, with the highest detectability in metastatic disease. Second, in a risk stratification setting, the ctDNA methylation panel discriminated metastatic disease from locally advanced disease with adequate accuracy. Notably, the diagnostic performance of PSA in the metastatic setting improved significantly when combined with the ctDNA methylation panel, suggesting that ctDNA methylation signals may capture biological features associated with advanced systemic disease beyond PSA alone. Third, the ctDNA methylation panel showed moderate performance in discriminating localised disease managed with AS from more advanced disease requiring active management (RP and mCSPC). Fourth, the ctDNA methylation panel demonstrated robust discrimination between mCSPC and controls but had limited ability to distinguish localised/locally advanced PCa from controls. Exploratory multivariable analyses further suggested that ctDNA methylation panel positivity remained associated with metastatic disease after adjustment for established clinicopathological variables.

The detectability of the ctDNA methylation panel followed a clear stage-dependent pattern, increasing from localised to metastatic PCa, consistent with differences in tumour burden and ctDNA shedding. This aligns with Bjerre et al., who demonstrated higher detectability of methylation-based ctDNA markers in metastatic compared with localised PCa [20]. While PCa-specific methylation alterations are present in tumour tissue from early-stage disease, their limited detectability in plasma highlights the sensitivity constraints of ctDNA assays at low tumour burden.

*DOCK2* and *HAPLN3*, previously investigated by Bjerre et al. [20], were included in our multiplex panel and showed a similar stage-dependent detection pattern despite differences in assay design, supporting their role as PCa-associated methylation signals. *ACTRT2*, *EVX1*, and *HOXD13* were selected based on conserved prostate-specific methylation patterns across benign and malignant prostate tissue and were included to improve assay sensitivity [24]. Notably, all five markers displayed a similar stage-dependent increase in detection across the disease spectrum. This concordant pattern suggests that the observed circulating methylation signal is associated with disease stage and tumour burden. Although contributions from adjacent non-malignant prostate tissue cannot be excluded, the similar behaviour of both tissue-conserved and cancer-associated markers supports a disease-related origin of the detected circulating DNA. The limited detectability of ctDNA in early-stage PCa is consistent with previous studies showing that plasma-based assays perform robustly in metastatic disease but remain challenged in localised stages due to low ctDNA levels [25]. As epigenetic alterations often occur early during carcinogenesis, their detectability in plasma remains strongly dependent on disease stage and overall ctDNA burden [26,27]. Accordingly, these findings support the biological validity of the assay and underscore the importance of disease stage when interpreting ctDNA-based biomarkers.

Additionally, this study investigated whether methylation markers detected by our multiplex ddPCR assay could discriminate between the four different cohorts in a diagnostic setting. The ctDNA methylation panel demonstrated moderate-to-high discrimination between patients with mCSPC and controls, whereas discrimination between localised/locally advanced PCa and controls was limited. Methylated ctDNA was detectable in the majority of patients with mCSPC, consistent with Bjerre et al., who reported detection in approximately two-thirds of metastatic cases, increasing to 89.3% on high-volume disease [20]. As nearly three-quarters of patients with mCSPC in our cohort had high-tumour volume, detection rates were comparable, supporting consistent ctDNA detectability across studies in patients with advanced disease burden. The use of a multi-locus panel and multiplex ddPCR likely increased analytical sensitivity in advanced disease, while individual methylation markers retained high specificity. Detection remained low in localised disease, with a modest difference between patients managed with RP and those undergoing AS, although the consistent direction of the association may suggest an association between detectable ctDNA and biological features linked to more aggressive disease behaviour in a subset of patients with organ-confined disease. Methylation positivity among controls should be interpreted in the context of both the multiplex panel design and the characteristics of the control cohort. Importantly, controls were not healthy volunteers but men undergoing diagnostic evaluation for suspected prostate cancer, primarily due to elevated PSA levels and/or clinical suspicion. Accordingly, the control cohort represents a clinically relevant pre-biopsy population with biological overlap with early PCa populations. While the combined five-marker panel was positive in 10.8% of controls, marker-specific positivity ranged from 0% to 4.6%, and no positive controls were observed for *HOXD13* or *DOCK2*. The observed panel positivity rate likely reflects the cumulative effect of integrating multiple markers to maximise sensitivity at low ctDNA concentrations across heterogeneous disease presentations, resulting in an inherent trade-off between sensitivity and specificity. Furthermore, no individuals in the control cohort had developed PCa at the latest available follow-up (December 2024; median follow-up 6.4 years, range 5.2–7.9 years). While longer follow-up may identify additional cases, these data suggest that subsequent PCa diagnoses are unlikely to explain the majority of positive findings observed in the control group. All samples were analysed in duplicate, supporting analytical reproducibility. However, without paired leukocyte DNA, the biological source of methylation signals in controls cannot be definitively established. Potential explanations include low-level background methylation and age-related methylation changes. Future studies incorporating paired leukocyte DNA may help clarify the origin of low-level methylation signals detected in control samples. These findings are consistent with the generally low abundance of ctDNA in early-stage disease. Clinically, the ctDNA methylation panel is unlikely to serve as a standalone diagnostic test in early disease or pre-biopsy settings. Its role in early diagnosis or biopsy triage, therefore, appears limited based on the present data. However, it may provide complementary information alongside existing diagnostic tools.

In a risk stratification context, the ctDNA methylation panel showed moderate discrimination between patients managed with AS and those receiving active treatment (RP or mCSPC), primarily driven by metastatic disease, whereas separation between AS and RP was limited. The biologically heterogeneous study groups, particularly the marked differences in baseline clinicopathological characteristics between localised, locally advanced, and metastatic disease, likely contributed substantially to the observed discriminatory performance. This reflects low ctDNA abundance in localised PCa, constraining discrimination within early-stage disease. Detectable ctDNA in localised disease may indicate biologically aggressive tumour clones and underlying heterogeneity not captured by standard clinicopathological factors. Consistent with this, preoperative ctDNA detection has previously been associated with increased risk of biochemical recurrence and metastatic progression [28]. However, the present study did not include longitudinal recurrence or survival analyses, and the biological and clinical implications of detectable ctDNA in localised disease therefore remain uncertain. Clinically, methylation markers may, therefore, provide complementary information for risk stratification, although prospective validation is required. Consistent with this, exploratory multivariable analyses demonstrated that ctDNA methylation panel positivity remained associated with metastatic disease after adjustment for PSA, ISUP grade, and T-stage and significantly improved model fit beyond these established clinicopathological variables. Although these findings should be interpreted cautiously given the exploratory nature of the analyses and the modest sample size, they suggest that the ctDNA methylation panel may provide information not fully captured by conventional clinicopathological factors. However, residual confounding by disease burden cannot be excluded. In discriminating between locally advanced PCa and metastatic disease, the ctDNA methylation panel demonstrated moderate performance. Discrimination improved significantly when the ctDNA methylation panel was combined with PSA compared to PSA alone. This suggests that ctDNA captures features associated with systemic disease burden beyond PSA alone. Thus, detectable ctDNA in patients with locally advanced disease may reflect biological features associated with increased systemic disease burden, although the potential prognostic and clinical implications remain to be established.

This study has several strengths. The cohort comprises the full spectrum of men suspected of PCa, within a tax-funded healthcare system with standardised diagnostic pathways, reducing selection bias and supporting representativeness. Plasma samples were collected prospectively with comprehensive clinical data, reducing information bias. The five-marker multiplex ddPCR assay demonstrated high analytical sensitivity and scalability for clinical laboratories. Few studies have evaluated multi-locus methylation panels in a representative diagnostic cohort. Limitations should be acknowledged. The study was conducted before the widespread implementation of multiparametric MRI and advanced imaging modalities (e.g., PSMA PET), limiting comparability with contemporary cohorts. As a single-region, multicentre study, generalisability may be limited. Subgroup sizes were modest. Although assay-specific limits of blank were predefined based on previous assay development work, external clinical validation of the ctDNA methylation panel in independent cohorts and laboratory settings remains necessary. Occasional low-level methylation signals among biopsy-negative controls underline the need for further marker panel optimisation. Although exploratory multivariable analyses suggested that the ctDNA methylation panel may provide information beyond established clinicopathological variables, larger studies are needed to validate these findings and determine their clinical relevance.

## 4. Materials and Methods

### 4.1. Study Design and Population

This exploratory diagnostic accuracy study included patients prospectively recruited to the PerPros prostate biobank. Patients were referred between September 2015 and December 2023 to the urology departments at Vejle, Esbjerg, and Fredericia hospitals for evaluation of suspected PCa, primarily due to elevated PSA levels.

Four groups were pre-specified: (i) biopsy-negative controls with no subsequent PCa diagnosis, (ii) men on active surveillance (AS) for low-risk PCa, (iii) men treated with radical prostatectomy (RP) for locally advanced PCa, and (iv) men with de novo metastatic castration-sensitive PCa (mCSPC). We planned to include 50 men per group in the first three groups and 100 men with de novo mCSPC. Slightly higher numbers were enrolled to compensate for potential assay failures. To minimise selection bias, consecutively enrolled patients were included. Blood samples were collected at the initial hospital visit prior to biopsy and treatment. Eligible participants were ≥18 years, with complete clinical data and no prior malignancies. The AS group fulfilled European Association of Urology low-risk criteria [5] (International Society of Urological Pathology (ISUP) grade 1, cT1–2a, and PSA < 10 µg/L). The RP group included patients with pT3–T4 and/or pN1 disease. The mCSPC group comprised patients presenting with metastatic disease without prior treatment or neuroendocrine/mixed histology. Controls had no histopathological evidence of PCa, no subsequent diagnosis during follow-up, and no high-grade prostatic intraepithelial neoplasia. Study data were managed using REDCap, an electronic data capture system hosted at the Open Patient data Explorative Network (OPEN), Odense University Hospital, Region of Southern Denmark [29].

### 4.2. Sample Collection, ctDNA Purification, and Multiplex ddPCR (ctDNA Analysis)

The methylation-specific multiplex droplet digital polymerase chain reaction (mm-ddPCR) assays were utilised as previously described by Kahns et al. [24]. Briefly, the mm-ddPCR assay targets six loci: two loci, *DOCK2* and *HAPLN3*, which are hypermethylated in PCa, and three loci, *ACTRT2*, *EVX1*, and *HOXD13*, which exhibit a conserved methylation pattern in both normal and cancerous prostate tissue, along with the *Albumin* reference gene. Plasma was isolated within 4 h of collection and stored at −80 °C according to standardised biobank protocols. Cell-free DNA was extracted from 4 mL plasma (the volume of three samples ranged between 2.5 and 3.9 mL) using the QIAsymphony DSP Circulating DNA kit (Qiagen, Hilden, Germany) and bisulfite converted using the EZ DNA Methylation Lightning kit (Zymo Research, Irvine, CA, USA) according to the manufacturer’s instructions. Assays were conducted on the QX600 ddPCR system (Bio-Rad Laboratories, Hercules, CA, USA). Samples were analysed in duplicate, including assay controls. Acceptance criteria are as described in Kahns et al. [24]. Droplet calling and absolute quantification were performed using QX Manager Software 2.0.0. Laboratory personnel were blinded to clinical data. The Limit of Blank (LoB) was determined as previously described [24].

### 4.3. Ethics Approval and Consent to Participate

Participants provided written informed consent for the use of blood samples and clinical data. The study was approved by the Regional Committees on Health Research Ethics for Southern Denmark (S-20240017) and the Danish Data Protection Agency (24/13038), in accordance with the Declaration of Helsinki.

### 4.4. Statistical Analysis

Descriptive statistics were applied to summarise patient characteristics, reported as medians with ranges (min; max) for continuous variables and proportions for categorical variables. Diagnostic performance of individual and combined methylation markers was assessed using receiver operating characteristic (ROC) analysis, reported as area under the curve (AUC) values and 95% confidence intervals (95% CI). Positive predictive value (PPV) and negative predictive value (NPV) were calculated at predefined cut-offs, based on assay-specific thresholds for ctDNA detection and clinically used thresholds for PSA. Logistic regression models were applied in a multivariate framework to evaluate the association between ctDNA methylation status and diagnostic categories. To explore whether the ctDNA methylation panel provided information beyond established clinicopathological variables, exploratory multivariable logistic regression analyses comparing mCSPC and localised PCa (AS and RP combined) were performed. Nested models were compared using likelihood ratio tests to evaluate the incremental contribution of ctDNA methylation panel positivity beyond PSA, ISUP grade, and T-stage. For multivariable analyses, Tx or unavailable T-stage values were categorised as “unknown” and retained in the model.

The discriminatory capacity of the ctDNA methylation panel and PSA, alone and in combination, was quantified using ROC analysis. Analyses were performed using STATA/BE 18.0 (STATACorp LLC, College Station, TX, USA), and a two-sided *p*-value < 0.05 was considered statistically significant.

## 5. Conclusions

This study demonstrates that the ctDNA methylation panel can effectively differentiate mCSPC from locally advanced PCa and, in this cohort, provides added discriminatory value beyond PSA alone. However, its clinical value remains limited in both diagnostic settings and in the early stages of PCa.

## Figures and Tables

**Table 1:** Baseline characteristics, *N* = 280.

		Control	AS	RP	mCSPC
		(*n* = 65)	(*n* = 55)	(*n* = 63)	(*n* = 97)
Age (median, min; max)		67 (40; 79)	65.5 (50; 73)	65 (51; 75)	72 (49; 87)
Family history of cancer					
	Yes	7 (10.8%)	10 (18.2%)	12 (19.1%)	5 (5.2%)
	No	17 (26.2%)	15 (27.3%)	20 (31.8%)	26 (26.8%)
	NA	40 (61.5%)	27 (49.1%)	30 (47.6%)	65 (67%)
	Missing	1 (1.5%)	3 (5.5%)	1 (1.6%)	1 (1%)
PSA (µg/L), baseline, median (min; max)		7.5 (1.5; 34)	6.5 (3.1; 9.3)	12 (4.5; 83)	84 (2.9; 9340)
PSA (µg/L) groups, *n* (%)					
	<10	45 (69.2%)	55 (100%)	26 (41.3%)	5 (5.2%)
	10–20	15 (23.1%)	0 (0%)	23 (36.5%)	11 (11.3%)
	>20	5 (7.7%)	0 (0%)	14 (22.2%)	81 (83.5%)
ISUP, *n* (%)					
	1		55 (100%)	4 (6.4%)	4 (4.1%)
	2		0 (0%)	29 (46%)	9 (9.3%)
	3		0 (0%)	19 (30.2%)	19 (19.6%)
	4		0 (0%)	6 (9.5%)	21 (21.7%)
	5		0 (0%)	5 (7.9%)	44 (45.4%)
T-stage, clinical, *n* (%)					
	T1–T2a		55 (100%)	38 (60.3%)	3 (3.1%)
	T2b		0 (0%)	9 (14.3%)	4 (4.1%)
	T2c		0 (0%)	8 (12.7%)	6 (6.2%)
	T3–T4		0 (0%)	7 (11.1%)	64 (66%)
	Tx		0 (0%)	0 (0%)	4 (4.1%)
	NA		0 (0%)	1 (1.6%)	16 (16.5%)
N-stage, clinical, *n* (%)					
	N0		10 (18.2%)	24 (38.1%)	40 (41.2%)
	N1		0 (0%)	1 (1.6%)	37 (38.1%)
	Nx		45 (81.8%)	37 (58.7%)	20 (20.6%)
	NA		0 (0%)	1 (1.6%)	0 (0%)
Metastases					
	Bone			0 (0%)	93 (95.9%)
	Lymph nodes			0 (0%)	29 (29.9%)
	Organ			0 (0%)	21 (21.7%)
Tumour volume ^					
	High				70 (72.2%)
	Low				27 (27.8%)
EAU risk groups, *n* (%)					
	Low risk		55 (100%)	1 (1.6%)	
	Intermediate risk		0 (0%)	44 (69.8%)	
	High risk		0 (0%)	18 (28.6%)	
T-stage, pathology *, *n* (%)					
	T1–T2a			1 (1.6%)	
	T2b			0 (0%)	
	T2c			6 (9.5%)	
	T3–T4			56 (88.9%)	
	Tx			0 (0%)	
N-stage, pathology *, *n* (%)					
	N0			23 (36.5%)	
	N1			23 (36.5%)	
ISUP, pathology *, *n* (%)					
	1			0 (0%)	
	2			28 (44.4%)	
	3			24 (38.1%)	
	4			3 (4.8%)	
	5			8 (12.7%)	

Percentages do not always add up to 100% due to data rounding. ^: Tumour volume defined by the CHAARTED study; *: pathology after radical prostatectomy; AS: active surveillance; EAU: European Association of Urology; ISUP: International Society of Urological Pathology; mCSPC: de novo metastatic castration-sensitive prostate cancer; NA: not available; N-stage: nodal stage; PSA: prostate-specific antigen; RP: radical prostatectomy; T-stage: tumour stage.

**Table 2:** Diagnostic performance of the individual ctDNA marker and the combined ctDNA panel.

ctDNA Marker	ctDNA Positive Controls	ctDNA Positive PCa	Sensitivity	Specificity	AUC	PPV	NPV
	(*n* = 65)	(*n* = 215)	(95% CI)	(95% CI)	(95% CI)	(95% CI)	(95% CI)
*HOXD13*	0	83	38.6%	100%	0.69	100%	33%
			(32.1–45.5)	(94.5–100)	(0.66–0.73)	(95.7–100)	(26.5–40)
*HAPLN3*	2	89	41.4%	96.9%	0.69	97.8%	33.3%
			(34.7–48.3)	(89.3–99.6)	(0.65–0.73)	(92.3–99.7)	(26.7–40.5)
*EVX1*	3	88	40.9%	95.4%	0.68	96.7%	32.8%
			(34.3–47.8)	(87.1–99.0)	(0.64–0.72)	(90.7–99.3)	(26.2–40)
*ACTRT2*	2	80	37.2%	96.9%	0.67	97.6%	31.8%
			(30.7–44)	(89.3–99.6)	(0.63–0.71)	(91.5–99.7)	(25.4–38.8)
*DOCK2*	0	81	37.7%	100%	0.69	100%	32.7%
			(31.2–44.5)	(94.5–100)	(0.66–0.72)	(95.5–100)	(26.2–39.7)
Combined ctDNA Panel	7	112	52%	89%	0.71	94.1%	36%
			(45.2–58.9)	(79.1–95.6)	(0.66–0.76)	(88.3–97.6)	(28.6–44)

AUC: area under the curve; CI: confidence interval; ctDNA: circulating tumour DNA; NPV: negative predictive value; PCa: prostate cancer; PPV: positive predictive value.

## Data Availability

The data generated and analysed during the current study are available from the corresponding author upon reasonable request, subject to approval by the PerPros biobank and relevant ethical authorities.

## Supplementary Materials

The following supporting information can be downloaded at: https://www.mdpi.com/article/10.3390/ijms27136081/s1.
