# Supplementary material for: Evaluation of a Methylated Circulating Tumour DNA Panel for Detection and Disease Stratification in Prostate Cancer
Source: Int J Mol Sci. 2026 Jul 7;27(13):6081. doi: 10.3390/ijms27136081 (PMC13361746; doi:10.3390/ijms27136081)
Supplement: Supplementary file 1 [file ijms-27-06081-s001.zip › Supplementary_legends.pdf]

Supplementary legends

Figure S1. ROC analysis across cut-points defined by the number of positive methylated ctDNA markers.

Receiver operating characteristic (ROC) curve for detection of prostate cancer using cut-points defined by ≥1 to ≥5 positive methylated ctDNA markers. N = 280.
AUC: area under the curve; ctDNA: circulating tumour DNA.

Figure S2. Diagnostic performance of individual ctDNA markers and the combined marker panel.

Receiver operating characteristic (ROC) curves for detection of prostate cancer using individual ctDNA markers and the combined marker panel. N = 280.
ctDNA: circulating tumour DNA.

Figure S3. ROC analysis of PSA at different cut-offs and the combined ctDNA panel.

Receiver operating characteristic (ROC) curves for detection of prostate cancer using prostate-specific antigen (PSA) at cut-offs ≥5, ≥10, and ≥20 µg/L and the combined ctDNA panel. N = 280.
ctDNA: circulating tumour DNA; PSA: prostate-specific antigen.

Figure S4. Performance of the combined ctDNA panel across prostate cancer subgroups.

(A) ROC analysis for discrimination of metastatic castration-sensitive prostate cancer (mCSPC) and radical prostatectomy (RP) from active surveillance (AS). N = 160 (mCSPC + RP) vs 55 (AS).
(B) ROC analysis for discrimination of RP from AS. N = 63 (RP) vs 55 (AS).
AUC: area under the curve; AS: active surveillance; ctDNA: circulating tumour DNA; mCSPC: metastatic castration-sensitive prostate cancer; RP: radical prostatectomy.

Table S1. Sensitivity and specificity across cut-points defined by the number of positive methylated ctDNA markers.

Detailed report of sensitivity and specificity for detection of prostate cancer across cut-points defined by the number of positive methylated ctDNA biomarkers.
ctDNA: circulating tumour DNA; PCa: prostate cancer.

Table S2. Diagnostic performance of PSA at different cut-offs for detection of prostate cancer.

Sensitivity, specificity, and predictive values for prostate-specific antigen (PSA) at cut-offs of 5, 10, and 20 µg/L for detection of prostate cancer.
AUC: area under the curve; CI: confidence interval; NPV: negative predictive value; PPV: positive predictive value; PSA: prostate-specific antigen.

Table S3. Performance of the combined ctDNA panel in risk stratification.
Diagnostic performance of the combined ctDNA panel across clinical groups defined by disease status and management. N = 280 (controls: N=65, AS: N=55, RP: N=63, mCSPC: N=97).
AUC: area under the ROC curve; C: controls; ctDNA: circulating tumour DNA; mCSPC: de novo metastatic castration-sensitive prostate cancer; NPV: negative predictive value; PPV: positive predictive value; RP: radical prostatectomy; AS: active surveillance.
