# Supplementary figures and images for: Evaluation of a Methylated Circulating Tumour DNA Panel for Detection and Disease Stratification in Prostate Cancer
Source: Int J Mol Sci. 2026 Jul 7;27(13):6081. doi: 10.3390/ijms27136081 (PMC13361746; doi:10.3390/ijms27136081)

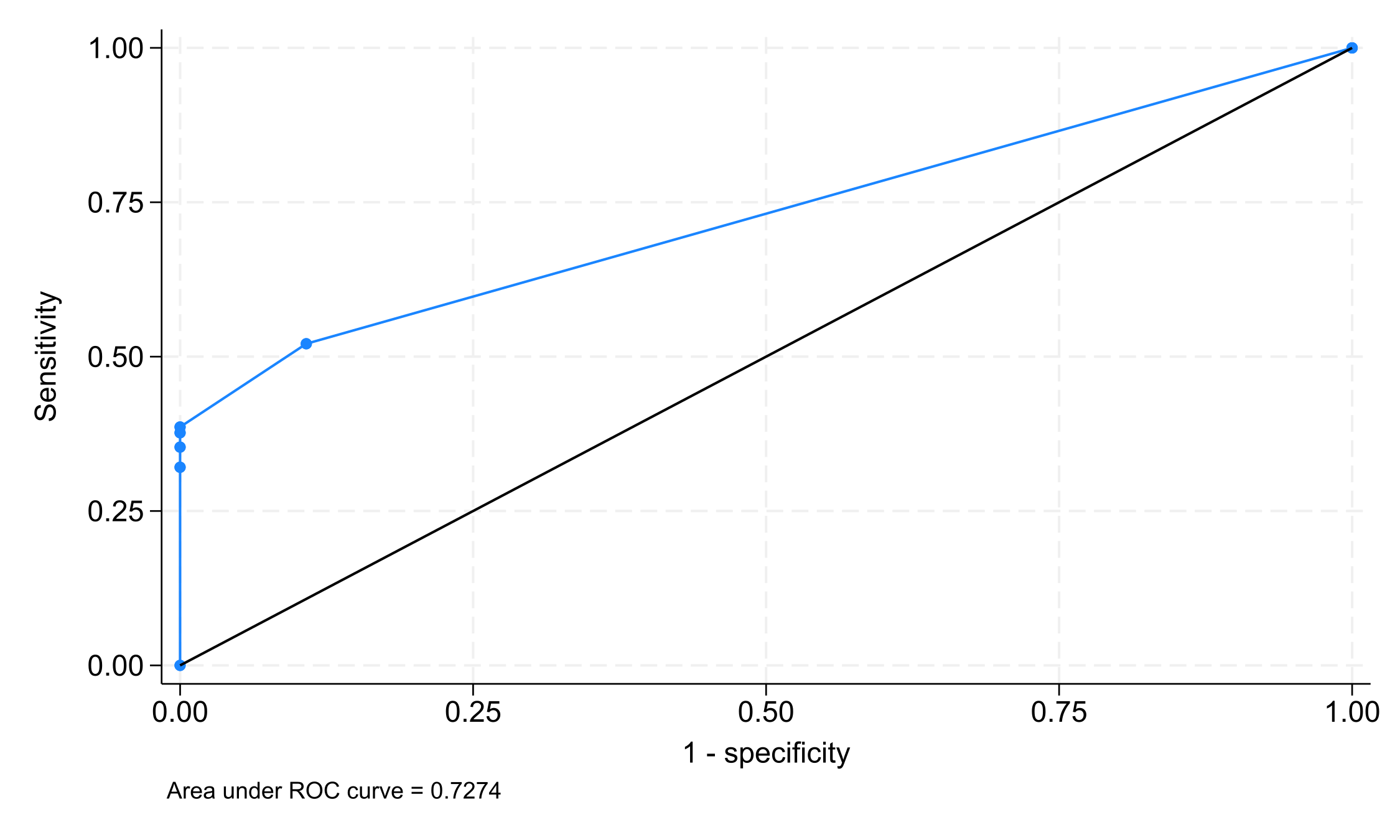

Supplement: Supplementary file 1 [file ijms-27-06081-s001.zip › Figure_S1.tiff]

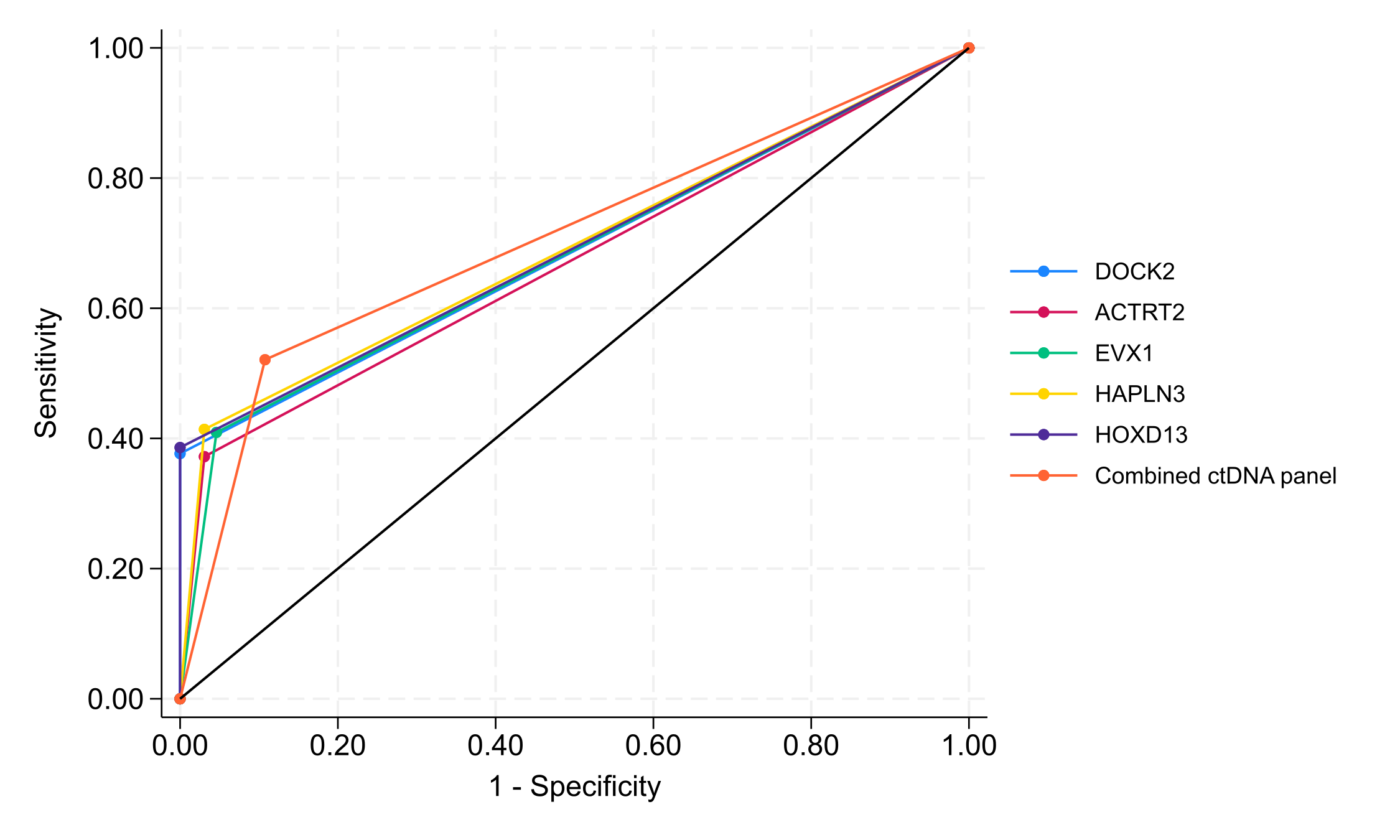

Supplement: Supplementary file 1 [file ijms-27-06081-s001.zip › Figure_S2.tiff]

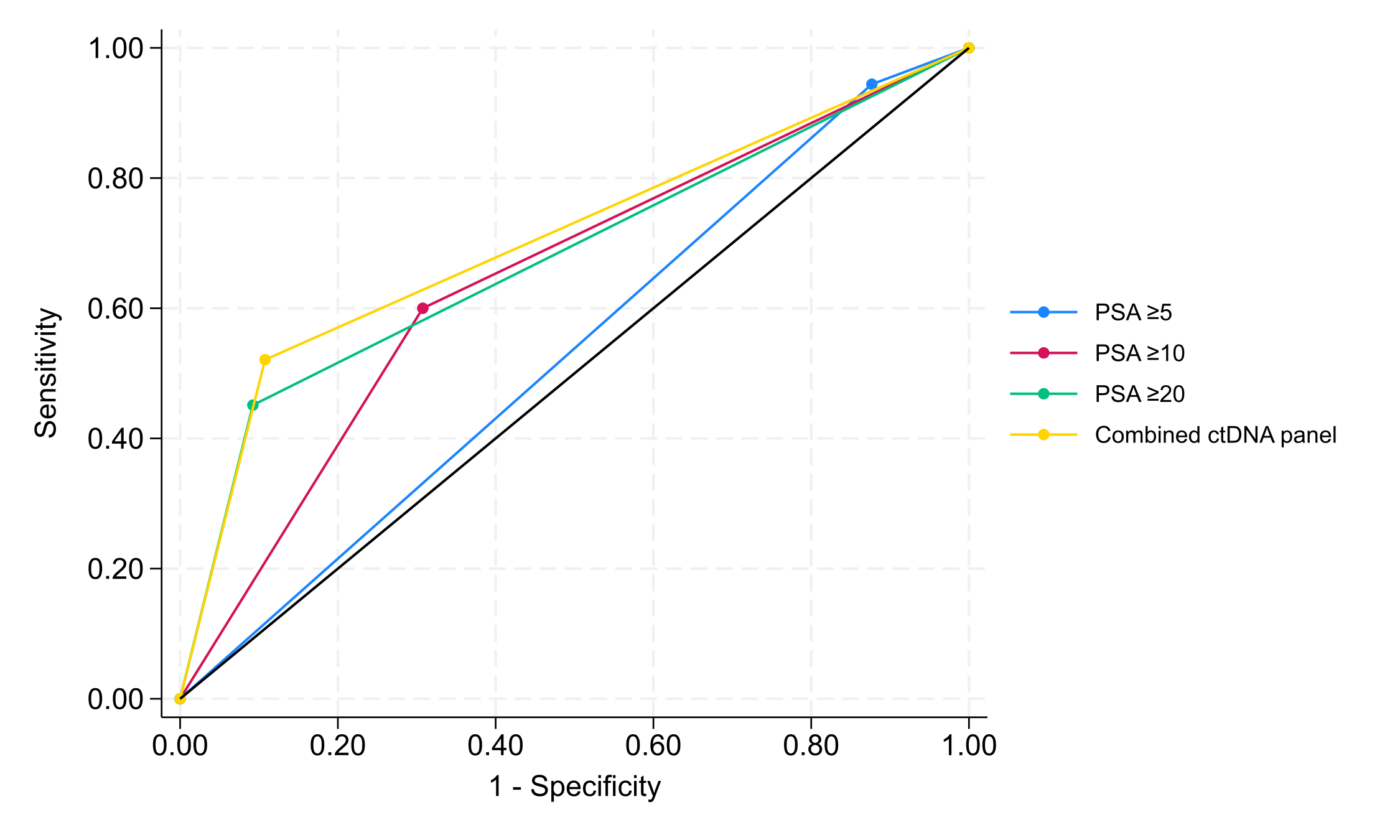

Supplement: Supplementary file 1 [file ijms-27-06081-s001.zip › Figure_S3.tiff]

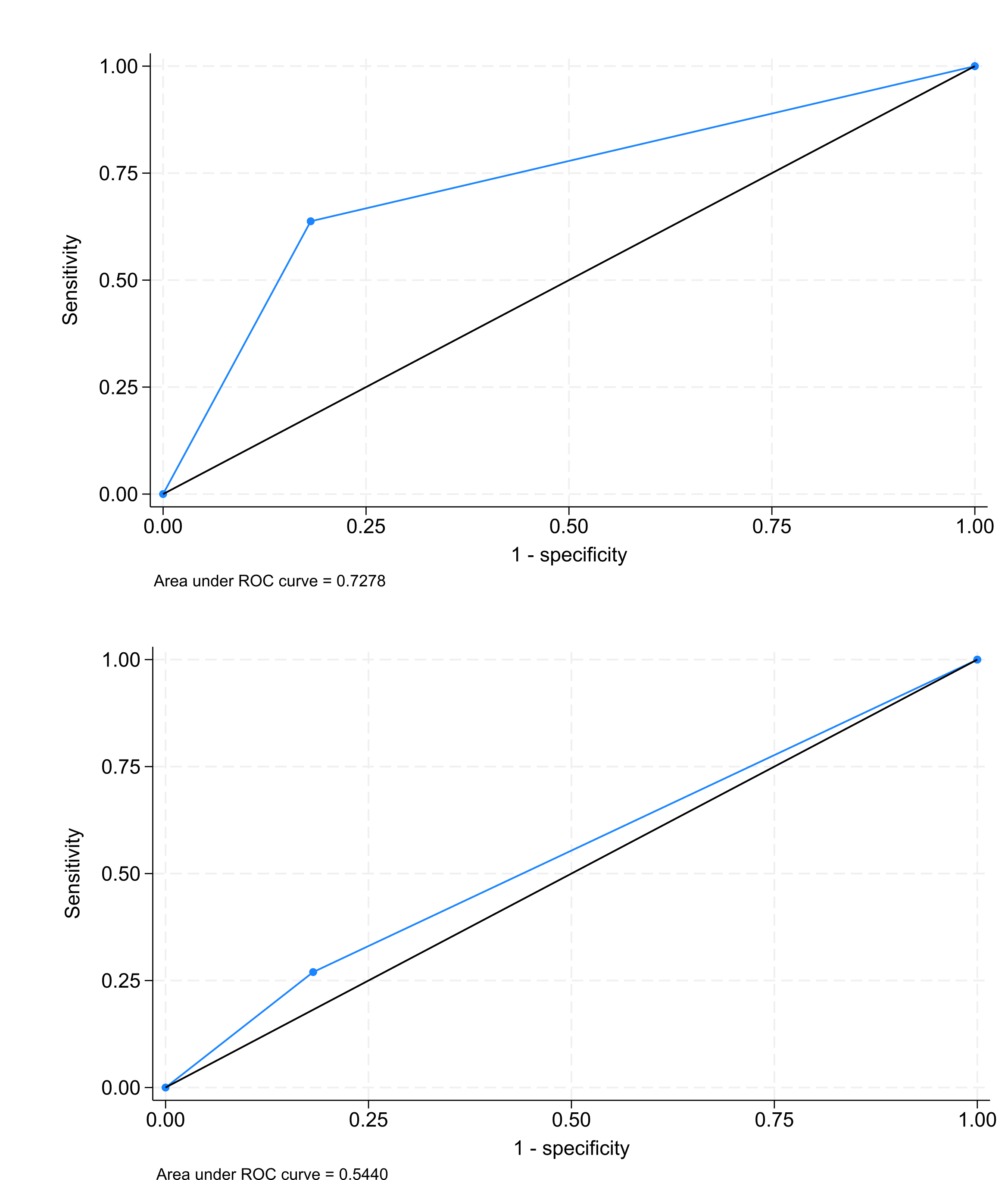

Supplement: Supplementary file 1 [file ijms-27-06081-s001.zip › Figure_S4AB.tiff]
